# Outcomes of coronoid-first repair through an anterior approach in patients with terrible triad injury of the elbow: a prospective study with a minimum 2-year follow-up

**DOI:** 10.1186/s10195-024-00804-z

**Published:** 2024-11-21

**Authors:** Wen-Chieh Chang, Ming-Fai Cheng, Kuei-Hsiang Hsu, Yu-Ping Su

**Affiliations:** 1 Department of Orthopaedics and Traumatology, Taipei Veterans General Hospital, No. 201, Sec. 2, Shipai Rd., Taipei, 11217 Taiwan; 2 Department of Orthopaedics, School of Medicine, National Yang Ming Chiao Tung University, Taipei, Taiwan

**Keywords:** Terrible triad injury, Elbow approaches, Elbow dislocation

## Abstract

**Background:**

In the treatment of terrible triad injury of the elbow (TTIE), the indication and the appropriate approach and sequence for coronoid process (CP) fixation remain debatable. No gold standard protocol has been established for CP fixation. In this study, we evaluated the midterm outcomes of coronoid-first repair through an anterior approach in patients with unstable TTIE.

**Materials and methods:**

This prospective observational study included patients with TTIE who exhibited posterior or posterolateral subluxation/dislocation during examination under anesthesia (EUA) at our institute between January 2019 and December 2021. All patients underwent coronoid-first repair through an anterior approach, regardless of fragment size. After CP fixation, radial head fixation/replacement and lateral ulnar collateral ligament repair were performed through the lateral Kocher approach. Radiographic and functional (Mayo Elbow Performance Score [MEPS] and Disabilities of Arm, Shoulder, and Hand score [DASH]) assessments were performed 3, 6, 12, and 24 months after surgery. Complications such as recurrent subluxation/dislocation, synostosis, heterotopic ossification, traumatic arthritis, and stiffness were examined at the follow-up visits.

**Results:**

The analysis included 27 patients. The mean follow-up duration was 29.9 (range 24–44) months. At the 3-, 6-, 12-, and 24-month follow-up, the mean flexion–extension arcs were 88.7° ± 14.7°, 107.9° ± 11.9°, 128.3° ± 15.5°, and 130.9° ± 15.3°; the mean supination–pronation arcs were 143.7° ± 9.9°, 160.4° ± 7.6°, 165.0° ± 6.0°, and 167.9° ± 4.9°; the mean DASH scores were 18.7 ± 5.7, 4.5 ± 6.1, 2.7 ± 6.5, and 2.0 ± 6.8; and the mean MEPS were 79.1 ± 10.3, 90.2 ± 8.3, 94.8 ± 6.6, and 95.9 ± 5.7, respectively. At the 24-month follow-up, 26 patients had excellent and 1 patient had good results according to MEPS. Only one patient had a complication: they exhibited stiffness and did not have a 30–130° flexion–extension arc at 24 months postoperatively.

**Conclusions:**

The EUA findings, rather than fragment size alone, may be a good indicator of whether the CP needs to be repaired. Midterm follow-up results implied that coronoid-first repair through an anterior approach yields satisfactory functional outcomes with minimal complications.

*Level of evidence*: Therapeutic level II.

## Introduction

Originally defined by Hotchkiss in 1996, terrible triad injury of the elbow (TTIE) refers to an injury pattern consisting of an elbow dislocation associated with a radial head fracture and a coronoid process (CP) fracture. As the elbow-dislocating force arises through a combination of forearm supination and valgus, the lateral ulnar collateral ligament (LUCL) is affected first. The force is then transmitted to the medial elbow, causing anterior and posterior joint capsule rupture and finally medial collateral ligament (MCL) injury [1, 2].

The anatomic relationships in TTIE have been extensively studied. The radial head contributes to valgus and external rotation stability, whereas the CP serves as a stabilizer against varus and internal rotatory stress [3–5]. In TTIE repair, the primary goal is to restore elbow stability by fixing the radial head whenever possible or replacing it with a prosthesis followed by repair of the LUCL [6]. However, the indication and the appropriate sequence for CP fixation remains the center of controversy in TTIE treatment [6–9].

Early studies indicated that systemic repair of the CP, regardless of the fragment size, and then successive repair of the radial head and LUCL led to an excellent functional outcome [10, 11]. However, this protocol was challenged by later cadaveric and clinical studies suggesting that the stability of the elbow is maintained when the fracture involves < 50% of the coronoid, and the instability only develops when the radial head is removed [7, 12–14]. Nonetheless, CP fracture is commonly associated with disruption of the anterior capsule, which can lead to instability, even in some coronoid tip fractures [6, 15, 16]. Some recent studies suggest that the repair of any coronoid fracture associated with elbow instability, regardless of the fragment size, yields a good outcome [6, 17, 18]. In addition to the indication and sequence, the approach used for CP fixation varies among surgeons. Protocols involving a single lateral or posterior incision and lateral combined medial or anterior approaches have all been described, each of which has its own advantages and disadvantages [6, 9–11, 19].

To date, it is still debatable whether the coronoid needs to be fixed and, if so, how, and in which sequence. There is no superior current protocol for treating these injuries [20–22]. Thus, the outcomes of coronoid-first repair through an anterior approach in patients with unstable TTIE were evaluated in the present study.

## Materials and methods

### Study design and cohort

This prospective observational study included patients at our institution between January 2019 and December 2021 who had an elbow dislocation associated with fractures of the radial head and CP. The inclusion criteria for final statistical analysis were defined as follows: patients underwent repair of the CP, radial head, and LUCL with a minimum of 2 years of follow-up. Exclusion criteria were (1) a trans-olecranon fracture dislocation, (2) a Monteggia fracture, (3) an associated upper-limb fracture at the ipsilateral side, or (4) an open fracture. The following data were collected: patient characteristics, injury mechanisms, fracture types, and fixation methods used for radial head and CP fractures. This study was approved by the institutional review board of our institution (no. 2019-09-013AC) and conducted in accordance with the ethical principles of the 1964 Declaration of Helsinki.

### Surgical protocol

Before surgery, computed tomography with three-dimensional reconstruction was universally performed to delineate the fracture pattern, comminution, and classification of the radial head and CP fracture. CP fractures were classified on the basis of the Regan–Morrey and O’Driscoll classification systems [23, 24], and radial head fractures were classified on the basis of the original Mason classification system because all such fractures would be classified as type IV in the Mason–Johnston system [25, 26]. In the original Mason classification system, type I indicates nondisplaced or small marginal radial head fractures, type II indicates partial articular fractures with > 2 mm of displacement, and type III indicates comminuted fractures involving the entire radial head.

**Fig. 1:**
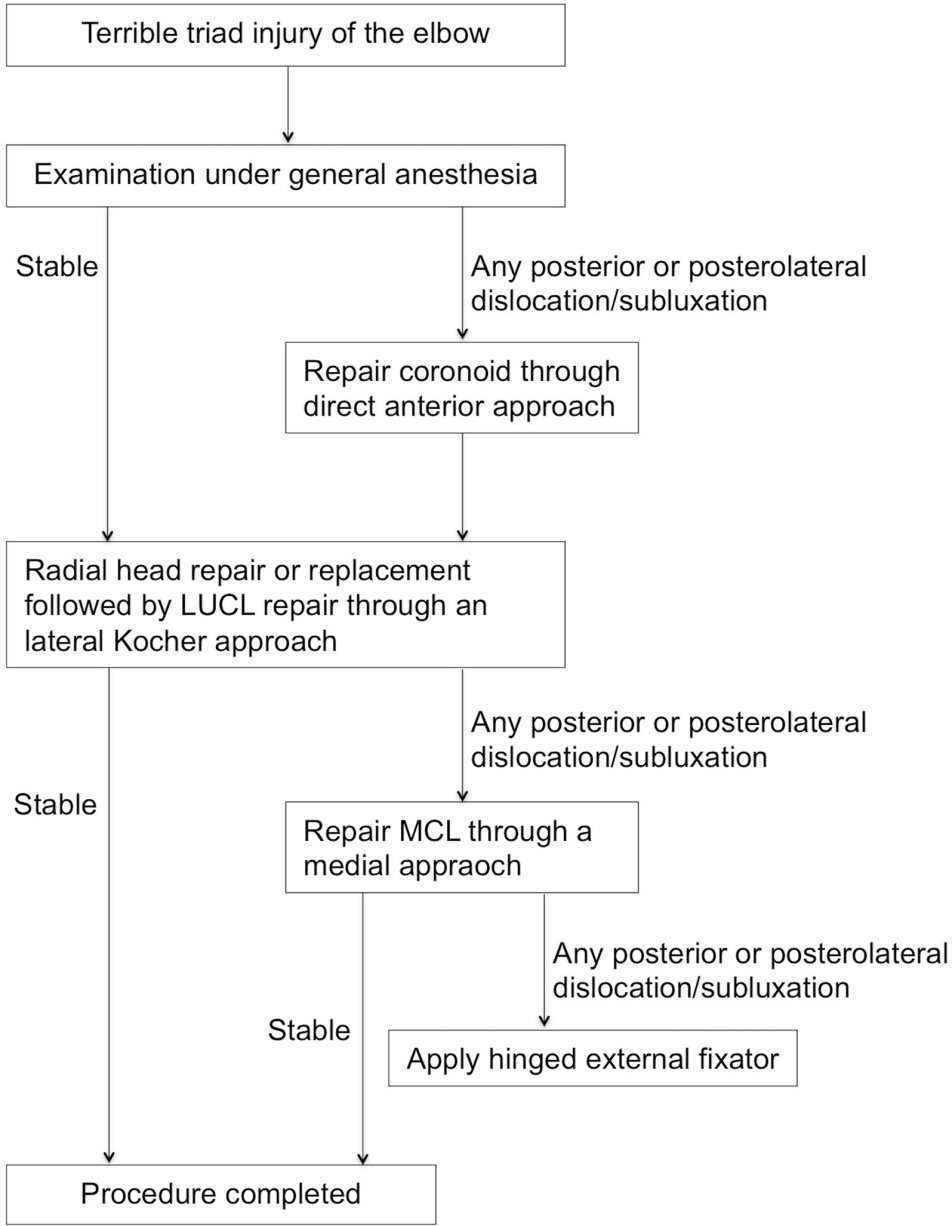
Protocol for operative treatment of terrible triad injuries of the elbow.* LUCL* lateral ulnar collateral ligament,* MCL* medial collateral ligament

**Fig. 2:**
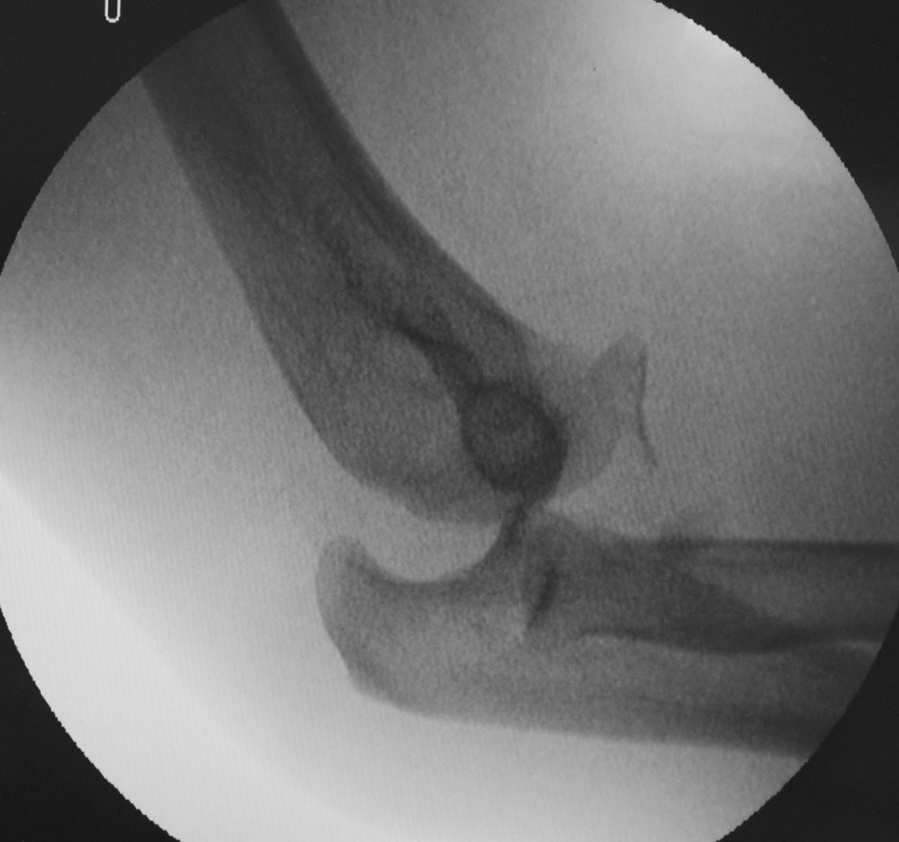
An example of instability defined by examination under anesthesia. A preoperative radiograph revealed posterior subluxation of the ulnohumeral joint during a test in a patient with terrible triad injury of the elbow

Figure 1 depicts our standard surgical protocol for TTIE. To assess elbow stability, an examination under anesthesia (EUA) was performed for all patients. A dorsally directed force relative to the humerus was applied to the forearm with the forearm in neutral rotation. Elbow instability was defined as the occurrence of any posterior or posterolateral subluxation/dislocation within a 20–130° flexion–extension arc (Fig. 2) [9].

For patients with a stable elbow, an isolated lateral Kocher approach was adopted for radial head repair or replacement and LUCL repair without CP fixation. By contrast, for those with an unstable elbow, a systematic CP repair was performed first through an anterior approach. Then, the radial head and LUCL were repaired by the Kocher approach. If instability persisted, the MCL was exposed through a medial incision and repaired. A hinged external fixator was used if patient had residual instability after MCL repair.

### Surgical technique

**Fig. 3:**
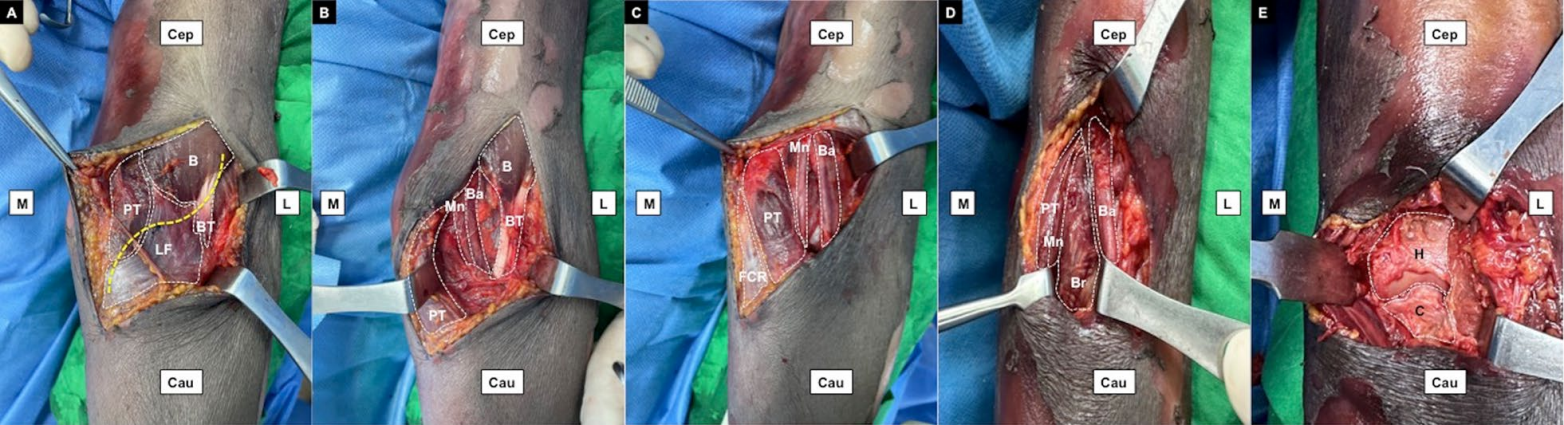
A cadaveric left elbow was utilized to illustrate our anterior approach for the coronoid process. **A** A curve incision (*dotted yellow line*) was made at the cubital fossa, starting from the proximoradial aspect of elbow flexion crease and extending to the distoulnar side of the elbow crease. After subcutaneous dissection, the bicep and its tendon, the pronator teres, and the lacertus fibrosus were exposed. **B** The lacertus fibrosus was transversely incised to reveal the underlying brachial artery and median nerve. **C** The interval between brachial artery and median nerve was created by mosquito forceps. **D** The brachial artery was retracted laterally, while the median nerve and pronator teres were retracted medially. The underlying brachialis was exposed.** E** The brachialis and its tendon was longitudinally incised. The capsule was opened and the whole coronoid process could be visualized.* B* biceps,* Ba* brachial artery,* Br* brachialis,* BT* biceps tendon,* C* coronoid process,* Cau* caudal,* Cep* cephalic,* FCR* flexor carpi radialis,* H* humerus,* L* lateral,* LF* lacertus fibrosus,* M* medial,* Mn* median nerve,* PT* pronator teres

**Fig. 4:**
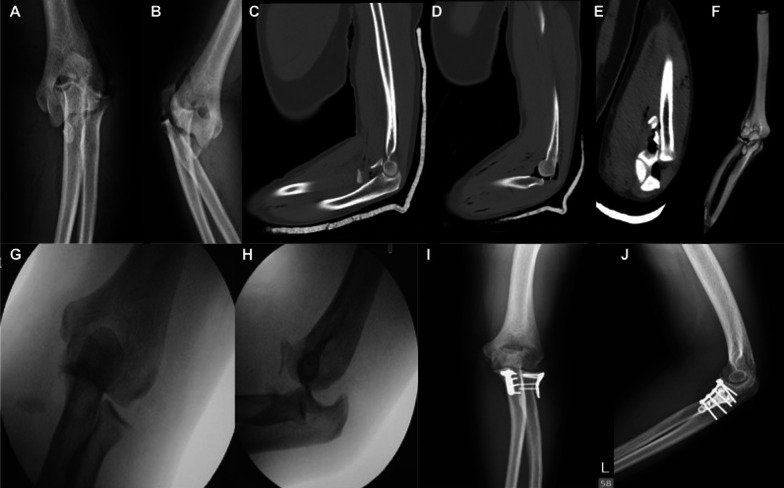
Case demonstration from our cohort. **A**, **B** Preoperative radiograph disclosed a 51-year-old patient with left terrible triad injury of the elbow; **C**–**F** computed tomography and three-dimensional reconstruction images revealed a Mason type II radial head fracture. The coronoid process fragment included the tip and anteromedial facet with less than 50% of the coronoid height (Regan–Morrey II) involved; **G**, **H** posterior subluxation of the ulnohumeral joint was observed during examination under anesthesia. **I**, **J** Radiograph at 2 years postoperatively. The patient first underwent coronoid process repair through an anterior approach. The radial head and lateral ulnar collateral ligament were addressed by the Kocher approach after coronoid process fixation

**Fig. 5:**
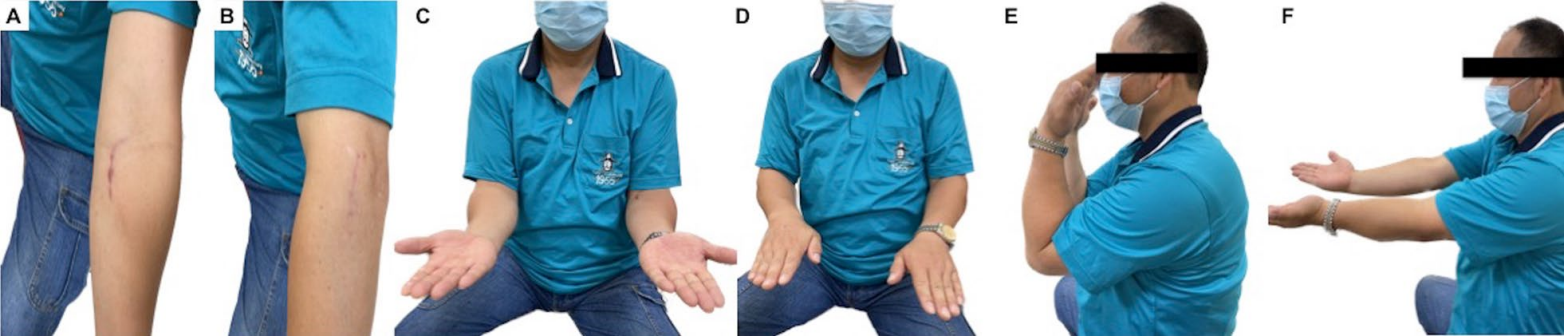
The patient in Fig. 4 at the 2-year follow-up. **A**, **B** Surgical wounds for the anterior and lateral approaches. The anterior incision can be much smaller in experienced hands. Only the ulnar portion of the S-shaped incision in Fig. 3A is required for adequate coronoid process exposure. **C**–**F** Forearm supination

All surgical procedures were performed by a single orthopedic trauma specialist. During the procedures, the patients were in the supine position and under general anesthesia. Our anterior approach for CP fixation is illustrated in Fig. 3. An S-shaped incision was made, starting from 2 cm proximal to the radial aspect of the elbow flexion crease, curving across the antecubital fossa, and ending at 2 cm distal to the ulnar side of the elbow crease. After subcutaneous blunt dissection, the cephalic vein, basilic vein, median cubital vein, and medial antebrachial cutaneous nerve were identified and retracted. Then, the pronator teres, biceps, and lacertus fibrosus were exposed. The lacertus fibrosus was transversely incised to reveal the brachial artery and median nerve. Unlike the traditional anterior approach, which involves the medial retraction of both the brachial artery and median nerve to disclose the brachialis, our approach involved creating an interval between the brachial artery and median nerve; this technique considerably reduced soft-tissue tension, further exposing the brachialis muscle. Our approach is theoretically safe because this space accommodates no neurovascular branches [27]. The brachial artery and biceps were laterally retracted, whereas the median nerve and pronator teres were medially retracted. A longitudinal incision was made at the brachialis and its tendon. The capsule was opened, exposing the entire CP fragment. After anatomic reduction under direct visualization, fixation with a 4.5-mm suture anchor was performed for a tip avulsion fracture, whereas a mini buttress plate, usually for a metacarpal or a 4.5-mm anteroposteriorly directed interfragment screw, was used for larger fragment. The lateral Kocher approach was used to address the radial head and LUCL. We preferred radial head fixation over replacement, even for Mason type III fractures. Ultimately, the LUCL was reattached to its anatomic origin on the lateral humeral epicondyle by using a 4.5-mm suture anchor. Figures 4 and 5 demonstrate a case in which our protocol and surgical technique for TTIE treatment was used.

### Postoperative management

Postoperatively, the patients received indomethacin (dosage: 25 mg thrice daily for 3 weeks) for heterotopic ossification prophylaxis. Celecoxib 200 mg once a day was given to relieve pain and allow early elbow rehabilitation. A supervised rehabilitation protocol was initiated at postoperative day 1 by our physical medicine and rehabilitation specialist. The patients performed functional exercises involving active elbow flexion and extension and forearm rotation. Active elbow flexion and extension and forearm rotation functional exercises lasting for 30 min were performed three times a day with a gradual range of motion increment. A hinged plaster splint was used for 8 weeks after the surgery. During the first 2 weeks, the elbow was immobilized at 90° flexion, with the forearm in neutral rotation outside the exercise period. For the next 6 weeks, the elbow flexion and extension parameters in the hinged splint were set as follows: 30–110° for 2 weeks, 20–120° for 2 weeks, and 10–130° for 2 weeks, followed by the full range of motion.

### Follow-up radiographic and clinical assessment

The patients underwent clinical and radiographic assessments at 1, 3, 6, 12, and 24 months postoperatively. Evidence of recurrent subluxation/dislocation, synostosis, heterotopic ossification, nonunion, implantation failure, and traumatic arthritis was documented from the follow-up radiograph. The elbow flexion and extension arc, range of forearm supination and pronation, and functional outcome as determined using the Mayo Elbow Performance Score (MEPS) and the Disabilities of Arm, Shoulder and Hand (DASH) score were assessed at the 3-, 6-, 12-, and 24-month follow-ups. The MEPS indicates elbow function based on measurements of pain, stability, movement, and daily activities. The total score is 100, and higher scores reflect better results, with greater than 90 being excellent, 75–89 good, 60–74 fair, and less than 60 poor. The DASH score (30 items rated on a scale of 1–5) rates the disability of the upper limb. Higher scores indicate poorer outcomes. In the current study, complications such as superficial and deep surgical wound infections and postoperative neuropathy were recorded at the follow-up visits. Stiffness was defined as the absence of a 30–130° flexion–extension arc and 100° of forearm rotation [28].

### Statistical analysis

The patients’ range-of-motion angles and functional scores were expressed in terms of the mean ± standard deviation. Statistical analyses were performed using SPSS (version 22; SPSS Statistics, IBM, Armonk, NY, USA).

## Results

During the study period, 33 patients with TTIE underwent CP fixation followed by radial head and LUCL repair at our institution. Of these patients, six were excluded for the following reasons: follow-up duration of < 24 months (*n  *= 3), concomitant fracture at the ipsilateral upper limb (*n* = 2), and open fracture (*n  *= 1). Finally, 27 patients were included in the analysis. The patients’ mean age at surgery was 52.3 ± 16.8 years, and the mean follow-up duration was 29.9 ± 5.6 months. Only one patient had elbow stiffness and failed to achieve a 30–130° flexion–extension arc at the 24-month follow-up. The characteristics of the patients are summarized in Table 1.

**Table 1:** Patient characteristics

Patient	Age (years)	Sex	Injury side	Injury mechanism	Follow-up (months)	Complication
1	38	M	R	Fall	26	–
2	39	M	R	Fall	25	–
3	52	M	L	Fall	34	–
4	84	M	R	Fall	32	Stiffness
5	18	F	R	Traffic accident	27	–
6	51	M	L	Traffic accident	44	–
7	18	F	L	Fall	25	–
8	48	M	R	Fall	26	–
9	50	M	L	Fall	31	–
10	54	F	L	Traffic accident	29	–
11	52	M	L	Fall	43	–
12	60	F	R	Fall	35	–
13	58	F	R	Fall	27	–
14	37	M	R	Fall	32	–
15	54	M	L	Fall	29	–
16	73	F	R	Fall	28	–
17	35	M	L	Traffic accident	27	–
18	65	F	R	Traffic accident	25	–
19	54	M	R	Fall	24	–
20	48	M	L	Fall	26	–
21	69	F	L	Fall	25	–
22	39	M	R	Fall	36	–
23	79	F	R	Traffic accident	41	–
24	72	F	R	Fall	29	–
25	33	M	R	Fall	27	–
26	67	F	R	Traffic accident	28	–
27	66	F	L	Fall	26	–
Mean ± SD	52.3 ± 16.8	–	–	–	29.9 ± 5.6	–

*SD* standard deviation

The classification of injury and treatment decision for each component are listed in Table 2. Regarding CP repair, all fixation methods, including an anteroposteriorly directed screw (*n* = 4), a suture anchor (*n  *= 8), and buttress plating (*n* = 15), were performed via an anterior approach in our cohorts, and were selected based on fragment size. For the radial head fracture, one patient underwent radial head replacement and one patient underwent conservative management for a minimally displaced Mason type I fracture. The remaining 25 patients underwent radial head fixation. All LUCL injuries in our patients underwent surgical repair with a 4.5-mm suture anchor. Only one patient underwent MCL repair due to intraoperative residual instability after fixation of the CP, radial head, and LUCL. No patient required hinge fixation.

**Table 2:** Classification of injury and treatment of each component

Patient	Radial head		Coronoid process			LUCL	MCL	Hinged fixator
	Mason	Treatment	Regan–Morrey	O’Driscoll	Treatment			
1	III	ORIF	I	Tip, sub1	SA	SA	–	–
2	II	ORIF	III	Basal, sub1	BP	SA	SA	–
3	II	ORIF	II	Tip, sub2	AS	SA	–	–
4	I	–	II	AM, sub2	BP	SA	–	–
5	II	ORIF	I	Tip, sub2	SA	SA	–	–
6	II	ORIF	II	AM, sub2	BP	SA	–	–
7	II	ORIF	II	Tip, sub2	BP	SA	–	–
8	II	ORIF	I	Tip, sub2	SA	SA	–	–
9	III	ORIF	II	AM, sub2	BP	SA	–	–
10	II	ORIF	II	Tip, sub2	AS	SA	–	–
11	II	ORIF	III	AM, sub3	BP	SA	–	–
12	II	ORIF	I	Tip, sub1	SA	SA	–	–
13	II	ORIF	II	AM, sub2	BP	SA	–	–
14	III	ORIF	II	AM, sub2	BP	SA	–	–
15	III	ORIF	I	Tip, sub2	SA	SA	–	–
16	II	ORIF	II	Tip, sub2	BP	SA	–	–
17	II	ORIF	I	Tip, sub2	SA	SA	–	–
18	II	ORIF	II	Tip, sub2	BP	SA	–	–
19	II	ORIF	III	Basal, sub2	BP	SA	–	–
20	II	ORIF	I	Tip, sub1	SA	SA	–	–
21	II	ORIF	II	Tip, sub2	AS	SA	–	–
22	II	ORIF	II	AM, sub2	BP	SA	–	–
23	III	RR	I	AM, sub1	SA	SA	–	–
24	III	ORIF	II	Tip, sub2	BP	SA	–	–
25	II	ORIF	II	Tip, sub2	AS	SA	–	–
26	II	ORIF	II	Tip, sub2	BP	SA	–	–
27	II	ORIF	II	AM, sub2	BP	SA	–	–

*AM* anteromedial,* AS* anteroposterior screw,* BP* buttress plate,* LUCL* lateral ulnar collateral ligament,* MCL* medial collateral ligament,* ORIF* open reduction and internal fixation,* RR* radial head replacement,* SA* suture anchor,* Sub* subtype

Table 3 presents the patients’ ranges of motion and functional outcomes at different follow-up visits. At the 3-, 6-, 12-, and 24-month follow-ups, the patients’ mean flexion–extension arcs were 88.7° ± 14.7°, 107.9° ± 11.9°, 128.3° ± 15.5°, and 130.9° ± 15.3°; their mean supination–pronation arcs were 143.7° ± 9.9°, 160.4° ± 7.6°, 165.0° ± 6.0°, and 167.9° ± 4.9°; their mean DASH scores were 18.7 ± 5.7, 4.5 ± 6.1, 2.7 ± 6.5, and 2.0 ± 6.8; and their mean MEPSs were 79.1 ± 10.3, 90.2 ± 8.3, 94.8 ± 6.6, and 95.9 ± 5.7, respectively. According to MEPS, 26 patients had excellent and one patient had good results at the 24-month follow-up.

**Table 3:** Ranges of motion and functional outcomes at different intervals (mean ± standard deviation)

	3 months	6 months	12 months	24 months
Flexion–extension arc (degrees)	88.7 ± 14.7	107.9 ± 11.9	128.3 ± 15.5	130.9 ± 15.3
Flexion contracture (degrees)	24.4 ± 7.5	17.8 ± 5.9	3.3 ± 8.7	3.0 ± 9.6
Supination–pronation arc (degrees)	143.7 ± 9.9	160.4 ± 7.6	165.0 ± 6.0	167.9 ± 4.9
Supination arc (degrees)	72.8 ± 5.9	80.4 ± 4.8	82.2 ± 3.8	83.9 ± 3.5
Pronation arc (degrees)	70.9 ± 4.4	80.0 ± 3.9	82.8 ± 3.8	84.1 ± 3.4
DASH	18.7 ± 5.7	4.5 ± 6.1	2.7 ± 6.5	2.0 ± 6.8
MEPS	79.1 ± 10.3	90.2 ± 8.3	94.8 ± 6.6	95.9 ± 5.7

*DASH* Disabilities of Arm, Shoulder and Hand,* MEPS* Mayo Elbow Performance Score

## Discussion

In this study, in patients with unstable TTIE, we first repaired the coronoid through an anterior approach and followed this by addressing the radial head and LUCL from a lateral approach, which yielded good functional results with a low complication rate at midterm.

To the best of our knowledge, no standard guidelines have been established for the surgical indications and sequence for CP repair in patients with TTIE [6–9]. The surgical protocol established by Pugh et al. and McKee et al. in 2004 and 2005, respectively, was regarded as the treatment standard for TTIE [10, 11]. Their protocol involves universal CP repair, regardless of fragment size, after which the radial head and finally the LUCL are addressed. This protocol led to excellent functional outcomes at the midterm follow-up. However, subsequent biomechanical and clinical studies have questioned the protocol’s efficacy. Evidence indicates that fractures involving < 50% of the coronoid (Regan–Morrey type I and II) can be effectively repaired without fixation. In patients with this type of fracture, elbow stability can be restored by repairing or replacing the radial head and reconstructing the LUCL complex [7, 12–14]. Consequently, many surgeons now opt to repair the radial head and LUCL first and then fix the CP if the fragment is large or if residual intraoperative instability is detected [2, 8, 29]. However, recent studies have revealed that despite the strong correlation between joint instability and the proportion of CP involvement, the elbow can be destabilized even in small CP fractures, including tip fractures [15–17]. We summed up the evidence reported in the literature and modified the aforementioned protocol. The decision to perform CP fixation should be based on the EUA results rather than fragment size alone. The CP should be repaired first if any instability is noted during the EUA. Our approach is based on the understanding that elbow instability may be overlooked if defined solely on the basis of fragment size. Most coronoid tip fractures are associated with disruption of the anterior capsule, and some patients with TTIE present with a concomitant MCL rupture or avulsion fracture, which further contributes to an unstable elbow. Thus, rather than relying on fragment size alone, the preoperative EUA results should be considered when deciding whether to perform CP repair in all patients with TTIE. Our findings support the notion that universal CP repair in patients with positive EUA results can lead to a stable elbow and promising functional outcomes. The coronoid-first repair approach may have the following advantages. First, in patients with positive EUA findings, reconstructing the ulnohumeral joint—the primary stabilizer of the elbow joint—can potentially enhance elbow stability compared with repairing the lateral component alone. Second, repairing the LUCL before achieving anatomic reduction of the ulnohumeral joint can lead to LUCL laxity or over-tensioning due to incomplete restoration of the normal ulnohumeral length [9, 10]. In the lateral-first protocol, the LUCL is temporarily fixed after radial head repair/replacement and then definitively repaired after CP fixation. The coronoid-first approach may streamline the LUCL repair process and minimize tensioning-related complications.

For CP fixation, researchers have described numerous surgical approaches, each with its own set of advantages and disadvantages. Surgeons who prefer a single incision can rely on the posterior or lateral incision approach. In the posterior (Boyd) approach, although both CP and radial head fractures can be repaired, a large surgical incision with substantial muscle detachment and dissection is generally required. Regarding the lateral approach, although it can yield favorable outcomes, it can be technically challenging in a real clinical setting [9, 30, 31]. Because soft-tissue swelling can occur in patients with acute trauma, surgeons may find it difficult to access the CP from a single lateral incision. Although the radial head can be excised to further expose the CP during radial head replacement, radial head fixation remains the preferred choice in most cases of TTIE. Furthermore, approaching the anteromedial facet of the CP through a lateral incision can be extremely challenging. Many studies have reported that adopting a medial approach with a lateral incision for CP repair can improve the postoperative range of motion and functional outcomes [6, 9, 18, 32, 33]. However, the medial approach requires extensive soft-tissue dissection and may still fail to ensure sufficient exposure, particularly for the lateral aspect of the coronoid process [19, 34]. An anterior approach can help visualize the entire CP and confirm anatomic reduction intraoperatively. The anterior approach has potential biomechanical advantages because implants providing direct buttress force can be easily applied through this approach. Furthermore, it requires only minimal soft-tissue dissection to achieve complete exposure of the CP. This approach preserves almost all elbow structures, including the wrist’s ulnar flexor, ulnar nerve, and ulnar collateral ligament. Traditionally, the anterior approach has been used to access the CP fragment through the medial retraction of both the brachial artery and median nerve. However, an interval between the brachial artery and median nerve to expose the CP was typically created in the present study. By creating this window and retracting the brachial artery laterally and the median nerve medially, a loose gap between these two structures is ensured; thus, only a gentle force is required for retraction. This technique is deemed safe because no branches of the median nerve or brachial artery are present in this space under normal anatomic conditions [27]. Although the literature suggests that the anterior approach facilitates rigid CP fixation and thus yields promising clinical outcomes, most findings have been derived from isolated CP fractures [27, 35–37]. In the present study, a protocol for guiding the indication and approach for CP repair in patients with unstable TTIE was established. The findings of this study indicate that the anterior approach can facilitate CP repair in patients with TTIE and yield favorable midterm functional outcomes.

Our study has some limitations. First, the sample size was relatively small. Second, all procedures were performed by a single surgeon at a single institution; thus, the results may not be reproducible. Finally, this study lacked comparison groups involving other approaches. Future large-scale comparative studies should be conducted to identify the optimal surgical approach for TTIE.

## Conclusions

In conclusion, the results of this study indicated that universally repairing the CP in patients with intraoperative instability achieves excellent functional results. For patients who require CP fixation in unstable TTIE, repairing the CP first through an anterior approach and following this by radial-head and LUCL repair yields a satisfactory prognosis and minimal complications at midterm.

## Data Availability

The datasets used and/or analyzed during the current study are available from the corresponding author on reasonable request.

## Abbreviations

CP: Coronoid process
DASH: Disabilities of Arm, Shoulder and Hand
EUA: Examination under anesthesia
LUCL: Lateral ulnar collateral ligament
MCL: Medial collateral ligament
MEPS: Mayo Elbow Performance Score
TTIE: Terrible triad injury of the elbow
